# Host CD39 Deficiency Affects Radiation-Induced Tumor Growth Delay and Aggravates Radiation-Induced Normal Tissue Toxicity

**DOI:** 10.3389/fonc.2020.554883

**Published:** 2020-10-22

**Authors:** Alina V. Meyer, Diana Klein, Simone de Leve, Klaudia Szymonowicz, Martin Stuschke, Simon C. Robson, Verena Jendrossek, Florian Wirsdörfer

**Affiliations:** ^1^Medical School, Institute of Cell Biology (Cancer Research), University of Duisburg-Essen, Essen, Germany; ^2^Department of Radiotherapy, University Hospital Essen, Essen, Germany; ^3^Departments of Medicine and Anesthesia, Beth Israel Deaconess Medical Center, Harvard Medical School, Harvard University, Boston, MA, United States

**Keywords:** ionizing radiation, purinergic signaling, CD73, cancer, ATP

## Abstract

The ectonucleoside triphosphate diphosphohydrolase (CD39)/5′ ectonuclotidase (CD73)-dependent purinergic pathway emerges as promising cancer target. Yet, except for own previous work revealing a pathogenic role of CD73 and adenosine in radiation-induced lung fibrosis, the role of purinergic signaling for radiotherapy outcome remained elusive. Here we used C57BL/6 wild-type (WT), CD39 knockout (CD39^−/−^), and CD73 knockout (CD73^−/−^) mice and hind-leg tumors of syngeneic murine Lewis lung carcinoma cells (LLC1) to elucidate how host purinergic signaling shapes the growth of LLC1 tumors to a single high-dose irradiation with 10 Gy *in vivo*. In complementary *in vitro* experiments, we examined the radiation response of LLC1 cells in combination with exogenously added ATP or adenosine, the proinflammatory and anti-inflammatory arms of purinergic signaling. Finally, we analyzed the impact of genetic loss of CD39 on pathophysiologic lung changes associated with lung fibrosis induced by a single-dose whole-thorax irradiation (WTI) with 15 Gy. Loss of CD73 in the tumor host did neither significantly affect tumor growth nor the radiation response of the CD39/CD73-negative LLC1 tumors. In contrast, LLC1 tumors exhibited a tendency to grow faster in CD39^−/−^ mice compared to WT mice. Even more important, tumors grown in the CD39-deficient background displayed a significantly reduced tumor growth delay upon irradiation when compared to irradiated tumors grown on WT mice. CD39 deficiency caused only subtle differences in the immune compartment of irradiated LLC1 tumors compared to WT mice. Instead, we could associate the tumor growth and radioresistance-promoting effects of host CD39 deficiency to alterations in the tumor endothelial compartment. Importantly, genetic deficiency of CD39 also augmented the expression level of fibrosis-associated osteopontin in irradiated normal lungs and exacerbated radiation-induced lung fibrosis at 25 weeks after irradiation. We conclude that genetic loss of host CD39 alters the tumor microenvironment, particularly the tumor microvasculature, and thereby promotes growth and radioresistance of murine LLC1 tumors. In the normal tissue loss of host, CD39 exacerbates radiation-induced adverse late effects. The suggested beneficial roles of host CD39 on the therapeutic ratio of radiotherapy suggest that therapeutic strategies targeting CD39 in combination with radiotherapy have to be considered with caution.

## Introduction

Radiotherapy (RT) alone or in combination with surgery and chemotherapy is a central component of curative or palliative treatment for many cancer patients. For example, patients suffering from advanced non–small cell lung cancer (NSCLC) receive standard treatment with fractionated RT to the thoracic region or concurrent platinum-based radiochemotherapy (RCT), yielding local control rates of 40–66% (1–3). Yet, intratumoral heterogeneity and high intrinsic or acquired radioresistance can lead to relapse, whereas a pronounced radiosensitivity of coirradiated normal lung tissue causes adverse effects in sensitive patients, thereby limiting the application of curative RT doses and therapy intensification efforts of RT or RCT (3, 4). Instead tolerable radiation doses are often linked to suboptimal tumor control, despite accepting side effects that decrease quality of life (2, 3, 5). Current efforts to improve RT outcome therefore aim at combining highly conformal RT with molecularly tailored treatments to increase efficacy of tumor cell killing or reduce adverse effects to normal tissues, respectively. Based on exciting findings about RT-induced support of local and systemic antitumor immunity particularly in combination with immunotherapy (6–11), further clinical trials evaluate the use of combining RT or RCT with immune checkpoint inhibitors (ICIs); yet despite encouraging results and durable responses obtained, for example, in NSCLC patients, only a fraction of patients respond to multimodal therapies with RCT and inhibitors of the PD-1/PD-L1 immune checkpoint, or patients develop resistance (12, 13). Because RT-induced immune effects can also contribute to radiation-induced adverse effects (14–16), sensitive patients may even suffer from more pronounced radiotoxicity or fear new immune-related adverse effects upon such multimodal combinatorial treatments (13, 17–19). A better understanding of the mechanisms underlying resistance to RT/ICI and of immune-associated adverse effects is required for the design of effective combinatorial treatments that are suited to overcome these limitations. For improving the therapeutic gain of RT, such combinatorial treatments will ideally sensitize the tumor cells only, or at least more substantially than the coirradiated normal tissues or prevent or reduce acute and late toxicities to normal tissues without protecting the tumor.

Herein, the purinergic pathway emerges as promising immune checkpoint for improving antitumor immune responses and the efficacy of immunotherapy in cancer patients, including lung cancer (20–24). The canonical adenosinergic pathway is an evolutionary conserved signaling system that converts extracellular proinflammatory ATP, a potent danger signal, into immunosuppressive adenosine (Ado) through the concerted action of ectonucleoside triphosphate diphosphohydrolase (CD39) and ecto-5′-nucleotidase (CD73), thus functioning as an “immune checkpoint” (25). CD39 and CD73 are expressed by immunosuppressive immune cell types, particularly regulatory T cells (Tregs) and regulatory B cells and mesenchymal stem cells, and are important to their immunosuppressive actions (25–27). Furthermore, CD39 and CD73 are expressed on other immune cells, for example, neutrophils, monocytes, macrophages, and dendritic cells, as well as on human alveolar and bronchial epithelial cells, fibroblasts and the vascular endothelium, and contribute in regulating their phenotypes (28, 29) and functions in various physiological processes with potential pathophysiological function, e.g., angiogenesis and vasculogenesis, barrier function, leukocyte transmigration, wound healing, and potentially cell death (30–32). Intriguingly, solid human tumors coopt the physiological functions of CD39 and CD73 to support tumor growth–promoting neovascularization, tumor metastasis, and tumor immune escape by various mechanisms (25, 33–38). In fact, CD39 and CD73 are expressed on various tumor cells supporting high extracellular Ado levels in the adverse microenvironment of solid tumors, particularly in the context of hypoxia (39). CD39 is overexpressed in lung cancer, and inhibition of CD39 on tumor cells alleviated their immunosuppressive activity (40). Furthermore, CD39 expression identified exhaustion of tumor-infiltrating CD8^+^ T cells in tumor regions of the lung (41). CD73 is also expressed on lung cancer cells, as well as on tumor-promoting mesenchymal stromal cells and myeloid-derived suppressor cells in tumor tissue from NSCLC patients, and has been identified as a prognostic factor for poor overall survival and recurrence-free NSCLC survival (42–44).

Thus, modulating CD39, CD73, and/or Ado in the tumor microenvironment (TME) is considered as attractive novel therapeutic strategy to limit tumor progression and improve antitumor immune responses (20, 45–47). Although up-regulation of CD39 and CD73 in circulating immune cells of cancer patients upon RCT has been reported (48), the role of the CD39/CD73 immune checkpoint for the tumor response to RT has not yet been investigated in detail. We hypothesized that activation of purinergic signaling may not only contribute to an immunosuppressive environment in NSCLC patients but also dampen RT-induced antitumor immune responses. Even more important, own previous work on the pathogenic role of CD73 in radiation-induced lung fibrosis in mice (49, 50) suggests that pharmacologic strategies inhibiting CD73-dependent accumulation of Ado might be suited to improve the therapeutic ratio of RT by influencing both tumor-promoting and fibrosis-promoting effects of CD73/Ado signaling in irradiated tumor and normal tissues. Yet, the role of CD39 in radiation-induced lung fibrosis had not yet been investigated.

The previously described canonical pathway produces Ado through the metabolism of ATP toward AMP through the action of CD39. Among the extracellular nucleotides that can highly increase and become catabolized under pathologic conditions is also the extracellular nicotinamide adenine dinucleotide (NAD^+^) in addition to ATP (51, 52). In this non-canonical pathway, extracellular NAD^+^ is metabolized into ADP ribose (ADPR) via the cyclic ADPR hydrolase (CD38). The product is further converted to AMP via the action of ectonucleotide pyrophosphatase/phosphodiesterase family member 1 (ENPP1 or CD203a/PC-1) (53–56). Moreover, CD203a is capable to hydrolyze ATP, NAD^+^, and ADPR directly to produce AMP, but CD203a has a (significantly) lower affinity to ATP (57, 58). Both described adenosinergic pathways are able to produce AMP that converges to CD73, where it is degraded to Ado. Thus, both the canonical and non-canonical pathway can exert functions in pathological settings.

Cancer and fibrosis are both complex multistep processes that are tightly integrated with deregulated immune defense. Because various cell types involved in the underlying wound healing and tissue repair mechanisms respond to both ATP and Ado (32), we were interested to further elucidate how host purinergic signaling, especially the canonical pathway, shapes radiation responses in tumor and normal lung tissues and to shed light on potential context-dependent actions of the purinergic signaling under malignant vs. benign conditions. Here, we used mice with genetic deficiency of CD39 and CD73 to explore the impact of CD39 and CD73 in the tumor host on radiation-induced tumor growth delay in the syngeneic LLC1 murine lung cancer model and the consequences of CD39 deficiency for radiation-induced lung fibrosis. A better understanding of the mechanisms underlying the beneficial and adverse effects of purinergic signaling in tumor and normal tissue responses to RT will allow us to define rational strategies for exploiting the CD39/CD73 immune checkpoint to improve RT outcome.

## Materials and Methods

### Cells

The murine Lewis lung carcinoma cell line LLC1 was purchased from ATCC (Manassas, VA, USA) and was cultured in Dulbecco modified eagle medium (DMEM) with 10% fetal calf serum (FCS).

### Mice

Eight- to twelve-week-old C57BL/6 [wild-type (WT) controls], CD39-deficient (CD39^−/−;^ C57BL/6 background) (59) and CD73-deficient mice (CD73^−/−^; C57BL/6 background; kindly provided from Dr. Linda F. Thompson, Oklahoma Medical Research Foundation, Oklahoma City, OK, USA) (30) were bred and kept under specific pathogen-free conditions in the Laboratory Animal Facilities of the University Hospital in Essen. Mixed genders of each mice strain were used. All protocols were approved by the universities' animal protection boards in conjunction with the legal authority (LANUV Düsseldorf) according to German animal welfare regulations and by the Committee on the Ethics of Animal Experiments of the responsible authorities [Landesamt für Natur, Umwelt und Verbraucherschutz (LANUV), Regierungspräsidium Düsseldorf Az.84-02.04.2015.A518 and 84-02.04.2014.A351].

### Thorax Irradiation

Mice were anesthetized with 2% isoflurane, placed in holders and irradiated either with a single dose of 0 (sham control) or 15 Gy over their whole thorax [whole-thorax irradiation (WTI)] using a Cobalt-60 source (Phillips, Hamburg, Germany; 0.5 Gy/min) as described previously (60). This setup for WTI with the Cobalt-60 source using a single dose of 15 Gy induces a moderate fibrosis in C57BL/6 mice (49).

### Mouse Tumor Model

Mouse syngeneic tumors were generated by subcutaneous injection of 0.5 × 10^6^ LLC1 cells into the hindlimb of mice (total volume 50 μL) as previously described for prostate cancer (61, 62). As predefined single-dose irradiation for LLC1 hind-leg tumors from the literature (63–65), a dose of 10 Gy was confirmed for the growth retardation of LLC1 hind-leg tumors (set up experiments) and replicated to study potential additional effects of genetic modification or drug treatment. Animals of each experimental group received a single subcutaneous injection. For radiation (tumor volume: ~ 100 mm^3^), mice were anesthetized (2% isoflurane), and tumors were exposed to a single dose of 10 Gy ± 5% in 5-mm tissue depth (~1.53 Gy/min, 300 kV, filter: 0.5 mm Cu, 10 mA, focus distance: 60 cm) using a collimated beam with an XStrahl RS 320 cabinet irradiator (XStrahl Limited, Camberly, Surrey, Great Britain).

### Tissue Preparation for Paraffin Sections

Lungs and tumors were fixed in 4% Paraformaldehyde (PFA) in phosphate-buffered saline (PBS), pH 7.2, and placed in embedding cassettes. After dehydration in 70% ethanol, PFA-fixed tumors were processed using automated standard procedures and subsequent embedding in paraffin. Six-micrometer tissue sections were obtained with a Leica microtome and mounted on coated microscope slides.

### Immunohistochemistry and Histology

Paraffin-embedded tissue sections were hydrated using a descending alcohol series, incubated for 10 min in H_2_O_2_ and for 10 min in target retrieval solution (95°C) and further incubated for 30 min with blocking solution (2% goat serum/PBS). Permeabilized sections were incubated with primary antibodies overnight at 4°C. Antigen was detected with a peroxidase-conjugated secondary antibody and DAB staining (Dako, Agilent, Santa Clara, USA). Nuclei were counterstained using hematoxylin. Tissue sections were stained with hematoxylin and eosin and Masson Goldner trichrome (Carl Roth, Karlsruhe, Germany) as previously described (49, 66).

### Immunofluorescence

Immunofluorescence staining of paraffin-embedded lung tissue was performed using the Mouse on Mouse (M.O.M.™) Immunodetection Kit BASIC (Vector Laboratories) according to the manufacturer's instructions. Tissue sections were incubated with anti-CD34 (MEC 14.7; Santa Cruz Biotechnology, Dallas, TX, USA) antibody ON at 4°C. Anti-CD34 was detected using a secondary anti–mouse Alexa Fluor® 488 antibody (Invitrogen, Thermo Fisher Scientific, Waltham, MA, USA). Slides were further stained with DAPI.

### Semiquantitative Analysis of Lung Tissue Using Orbit Image Analysis

Osteopontin (OPN) and transforming growth factor β (TGF-β) (R&D Systems, Bio-Techne GmbH, Wiesbaden-Nordenstedt, Germany) stained lung tissue slides were photographed using a 25-fold magnification, and pictures were analyzed for positively stained areas in the lung tissue using the Software Orbit Image Analysis version 2.65 [Idorsia Pharmaceuticals Ltd., (67)]. Positively defined areas were calculated as percentage of the whole tissue lung section.

### Semiquantitative Analysis of the Tumor Proliferative Activity

DAB quantification of slides stained for proliferation cell nuclear antigen (PCNA) (GeneTex Inc., Irvine, USA) and counterstained with hematoxylin was performed by semiquantitative analyses. Ten random, non-overlapping fields (magnification, ×200) of tumor tissue from each specimen were photographed, and pictures were automated single cell counted for DAB using the “Fiji” version of ImageJ from http://fiji.sc (68). The following adjustments for automatic counting were used: Color Deconvolution DAB, threshold 150, particle size 150–6,000, Circularity 0.14–1.00. From all fields, the mean counts were averaged to yield the final score for each specimen.

### Fibrosis Scoring

Lung sections were stained with hematoxylin and eosin (H&E) or Masson Goldner trichrome (MT) (Carl Roth) and scored by four individuals blinded to the genotype and treatment group. Ten random, non-overlapping fields (magnification, ×200) of lung parenchyma from each specimen were photographed, and lung fibrosis was scored using a 0–8-point *Ashcroft* scale (69). The mean scores for each observer were averaged to yield the final score for each specimen.

### Tumor-Infiltrating Immune Cell Phenotyping

Tumors were cut into pieces, and the tissue was sequentially passed with DMEM medium through a 70-μm cell strainer and subsequently centrifuged by 1,500 rpm for 7 min. Total tumor cells were then rinsed with an erythrocyte lysis buffer (containing 0.15 M NH_4_Cl, 10 mM KHCO_3_, and 0.5 M EDTA), passed through a 30-μm cell strainer, and washed with DMEM medium and 10% FCS for subsequent phenotyping. Isolated cells were stained with fixable viability dye eFluor780 to identify living cells and anti–mouse CD45 PacificBlue (30-F11) for total leukocytes, respectively. Within tumor-infiltrating leukocytes, populations were further characterized for Ly6C, Ly6G, CD11b, CD11c, CD3, CD4, and CD8. Antibodies were obtained from BD Biosciences (Heidelberg, Germany), BioLegend (Fell, Germany), or eBioscience (Frankfurt, Germany). Analyses were performed on an LSRII using FACS DIVA Software version 8.0.1 (BD Biosciences, Germany).

### Irradiation of Cell Cultures

Radiation with a dose of 0, 5, and 10 Gy was performed using the ISOVOLT-320 X-ray machine (Seifert–Pantak, East Haven, CT) at 320 keV, 10 mA, dose rate about 3 Gy/min with a 1.65-mm aluminum filter, and a distance of about 500 mm to the object being irradiated (61).

### Treatment of Cells

For the flow cytometry analysis of the LLC1 cell line, cells were incubated with ATP used at a final concentration of 1,000 μg/mL and Ado at a final concentration of 2,000 μg/mL (both purchased from Sigma–Aldrich Chemie GmbH, Steinheim, Germany). These concentrations were chosen according to *in vitro* investigations for determination of the half maximal inhibitory concentration (IC_50_) using the crystal violet assay data at 72 h after treatment (70).

### Flow Cytometry Analysis of Cell Cultures

The mitochondrial membrane potential (ΔΨm) was analyzed using the ΔΨm-specific dye tetra-methyl-rhodamine ethyl ester (TMRE; Molecular Probes, Thermo Fisher Scientific, Grand Island, NY, USA). Cells were stained for 30 min in PBS containing 25 nM TMRE. For quantification of apoptotic DNA fragmentation (sub-G1 population), cells were incubated for 15–30 min with a staining solution containing 0.1% (wt/vol) sodium citrate, 50 μg/mL Propidium iodide (PI), and 0.05% (vol/vol) Triton X-100 (vol/vol) (61, 62). For quantification of expression of surface markers, cell lines were further fluorochrome-labeled with antibodies against CD73, CD39, P2X7R (Biolegend), AdoRA1 (Bioss Antibodies, Woburn, USA), AdoRA2A (Santa Cruz, Heidelberg, Germany), AdoRA2B (Bioss Antibodies), and AdoRA3 (Abcore, Ramona, USA). The specificity of all antibodies has been tested using primary murine total lung cells. The specificity of anti-CD73, anti-CD39, anti-AdoRA2A, and anti-AdoRA2B had been additionally tested using cells from the respective knockout mice (CD39, CD73, A2AR, A2BR) as a control. Cells were subsequently analyzed by flow cytometry using Cellquest Pro (FACS Calibur; Becton Dickinson, Heidelberg, Germany).

### Real-Time Reverse Transcription Polymerase Chain Reaction

RNA was isolated using RNeasy Mini Kit (74,106; Qiagen, Hilden, Germany) according to the manufacturer's instruction. Total RNA (1 μg) was used for reverse transcription with Superscript™-II reverse transcriptase (Qiagen) using oligo-dT primers according to the manufacturer's instructions; 0.5 μL of obtained cDNA was used for polymerase chain reaction (PCR) reaction as previously described (71). Analysis was carried out using the oligonucleotide primers (bActin_fw CCAGAGCAAGAGAGGTATCC, bActin_bw CTGTGGTGGTGAAGCTGTAG; VE-Cad_fw CAG CAC TTC AGG CAA AAA CA, VE-Cad_bw ATTCGGAAGAATTGGCCTCT; VEGFR2_fw ATGACAGCCAGACAGACAGT; VEGFR2_bw GGTGTCTGTGTCATCTGAGT, VCAM_fwAAGGCAGGCTGTAAAAGAATTG, VCAM_bw ATTCCAGAATCTTCCATCCTCA, CD38_fw ACAAGAGAAGACTACGCCCCAC, CD38_bw CACACCACCTGAGATCATCAGC, ENPP1_fw AGAACAGGACCACAGTGGGAAG, ENPP1_bw TAACAGGGGAAAGGGCAAGGAG) as previously described (72, 73).

### Statistics

Statistical analyses were performed using Prism 7.0e (GraphPad, La Jolla, CA, USA). Student two-tailed unpaired *t*-tests were used to compare differences between two groups. One-way analysis of variance (ANOVA) followed by Tukey multiple comparison tests were used to compare more than two groups. Two-way ANOVA with *post hoc* Tukey, Bonferroni, or Newman–Keuls multiple comparison tests were used to compare groups split on two independent variables. Statistical significance was set at *P* < 0.05.

## Results

### Loss of Host CD39 Abrogates Radiation-Induced Tumor Growth Delay, Whereas Loss of CD73 Had No Effect

**Figure 1:**
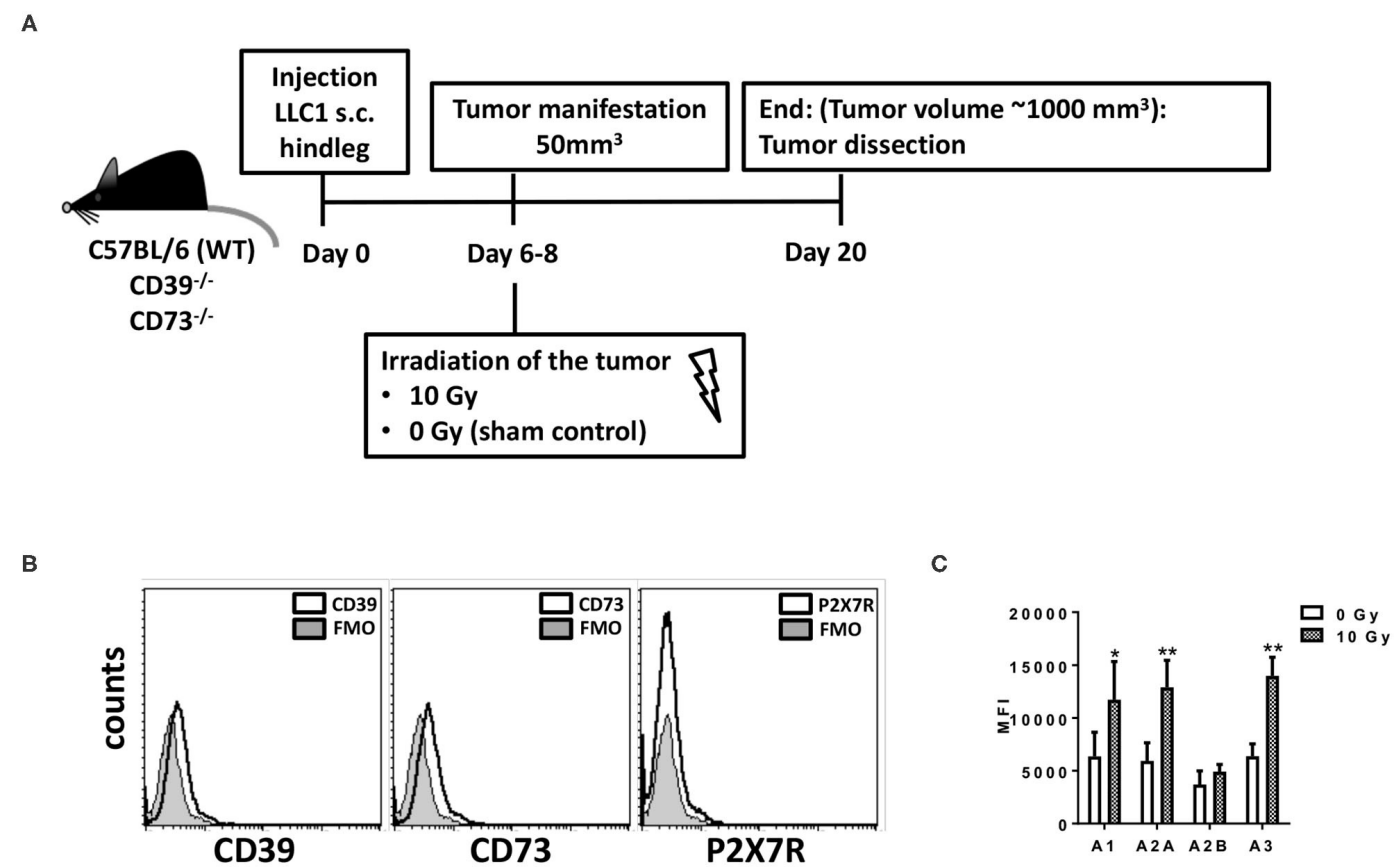
LLC1 murine lung cancer cells are deficient for CD39, CD73, and P2X7 but express the four Ado receptors. For *in vivo* experiments mouse syngeneic tumors were generated by subcutaneous injection of 0.5 × 10^6^ LLC1 cells into the hindlimb of C57BL/6 (WT), CD73^−/−^, and CD39^−/−^ mice. On days 6–8 after tumor manifestation, tumors were irradiated with 0 Gy (sham) or 10 Gy. Tumors were dissected at day 20 or when the tumor volume reached 1,000 mm^3^. **(A)** Schematic depiction of the experimental setup. **(B,C)** LLC1 cells were stained against **(B)** CD39, CD73, P2X7R; FMO = fluorescence minus one) or **(C)** AdoR- A1, A2A, A2B, and A3 and were further analyzed by flow cytometry (data show means ± SD; *P ≤ 0.05, **P ≤ 0.01 by one-way ANOVA followed by *post hoc* Newman–Keuls test).

**Figure 2:**
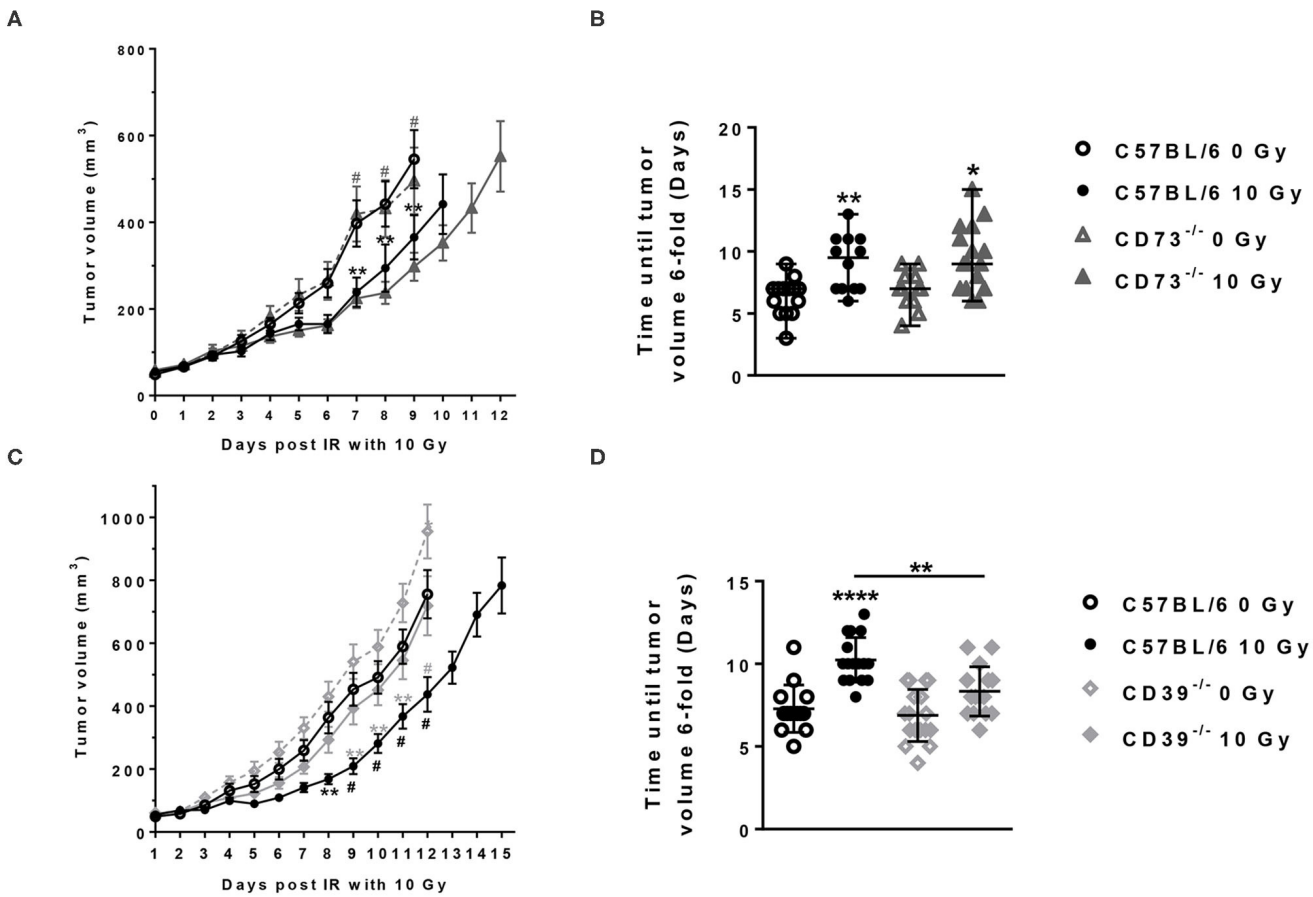
Host CD39 deficiency accelerates LLC1 tumor growth and reduces radiation-induced tumor growth delay. LLC1 tumor cells (0.5 × 10^6^ cells each) were subcutaneously transplanted onto the hindlimb of C57BL/6 wild-type (circles) CD73-knockout [knockout (^−/−^), triangles] and CD39^−/−^ (diamonds) mice. Hind-leg single-dose irradiation with 0 or 10 Gy was conducted at the timepoint of tumor manifestation (~100 mm^3^). LLC1 tumor volume in mm^3^ in respective experimental groups was determined at indicated time points using a sliding caliper (A/C). Growth delay was calculated as time (days) until the 6-fold tumor volume was reached (B/D). Shown are means ± SEM, *P ≤ 0.05, **P ≤ 0.01, ^#^,****P ≤ 0.0001 analyzed by two-way ANOVA followed by a *post hoc* Tukey test, *n* **(A)** = 15/17/17/15; *n* **(B)** = 17/15/13/16; n **(C)** = 16/14/13/16, *n* **(D)** = 13/14/15/15. In **(A,C)**, significant results between the animal of the same groups are marked with asterisks (0 and 10 Gy) and between the strains (C57BL/6 10 Gy and CD73^−/−^ or CD39^−/−^ 10 Gy) with hashtags.

First, we aimed to explore the role of CD39 and CD73 in the tumor host for tumor growth and response to therapeutic irradiation using Lewis lung carcinoma (LLC1) cells, a syngeneic C57BL/6 tumor model. LLC1 tumor cells were subcutaneously implanted onto the hind-leg of C57BL/6 WT mice, CD39-deficient mice (CD39^−/−^), or CD73-deficient mice (CD73^−/−^) (Figures 1, 2). When the tumors reached a volume of 50–100 mm^3^ (after 5–7 days), tumors of each mouse strain were irradiated with 0 Gy (sham control) or 10 Gy, and tumor growth was followed up until tumors reached a critical size of 1,000 mm^3^ (Figure 1A).

Characterization of purinergic signaling in the tumor cells revealed that LLC1 cells uncovered a CD39 gene expression that was not altered by irradiation (data not shown), but cell surface staining revealed that the LLC1 cells did neither express CD39, CD73, nor the ATP receptor P2X7R (Figure 1B), nor did irradiation with 5 or 10 Gy induce an expression of these markers (data not shown). However, LLC1 cells expressed all four AdoR, namely, AdoRA1, AdoRA2A, AdoRA2B, and AdoRA3, and except AdoRA2B, the expression levels of the AdoR further increased upon irradiation (Figure 1C and Supplementary Figure 1). Thus, the LLC1 cells were an optimal tool to investigate the role of CD39 and CD73 in the tumor–host on tumor growth and RT response.

No differences in growth were detected between tumors growing on the unirradiated C57BL/6 and CD73^−/−^ mice between the day of irradiation and the end of the experiment (Figures 2A,B). As expected, exposure to a single dose of 10 Gy induced a significant growth delay of the LLC1 tumors in the irradiated C57BL/6, as well as in the irradiated CD73^−/−^ mice, compared to their unirradiated controls resulting in a difference in tumor volume of 180.2 ± 47.75 mm^3^ in WT and 210.2 ± 47.63 mm^3^ in CD73^−/−^ mice at day 9 after irradiation (Figures 2A,B). Although there was a trend for a slight increase in growth retardation of irradiated LLC1 tumors grown on CD73-deficient background compared to WT mice, the differences in radiation-induced tumor growth delay between the LLC1 tumors grown in the C57BL/6 and CD73^−/−^ mice did not reach statistical significance (Figures 2A,B).

In contrast, tumors tended to grow faster on the CD39-deficient mice than on WT mice (Figure 2C). The follow-up of the tumor growth demonstrated that without irradiation tumors grown on CD39^−/−^ mice reached a certain tumor volume at an earlier time point than tumors grown on WT mice, yielding a significant difference of ~200 mm^3^ between WT and CD39^−/−^ mice at day 12 (Figure 2C). Exposure of the LLC1 tumors to a single irradiation with 10 Gy significantly delayed LLC1 tumor growth in both backgrounds (Figures 2C,D). Herein, average differences in tumor volumes of ~200 ± 100 mm^3^ between irradiated tumors and sham controls were obtained at days 8–12 in tumors grown on WT mice and were thus comparable to the growth delay values obtained in the experiments with WT and CD73^−/−^ mice described above (Figures 2A,B). In contrast, irradiated tumors grown on CD39^−/−^ mice showed a significantly reduced growth delay in comparison to the values observed in irradiated tumors from WT mice (Figures 2C,D). The time until the tumors reached a 6-fold volume upon irradiation was significantly shorter (by 2 days) for tumors growing on CD39^−/−^ mice compared to irradiated tumors on WT mice, indicating a radioresistance promoting effect of CD39 deficiency in the tumor host (Figure 2D). These differences were not due to differences in tumor volume at the time of irradiation (data not shown).

**Figure 3:**
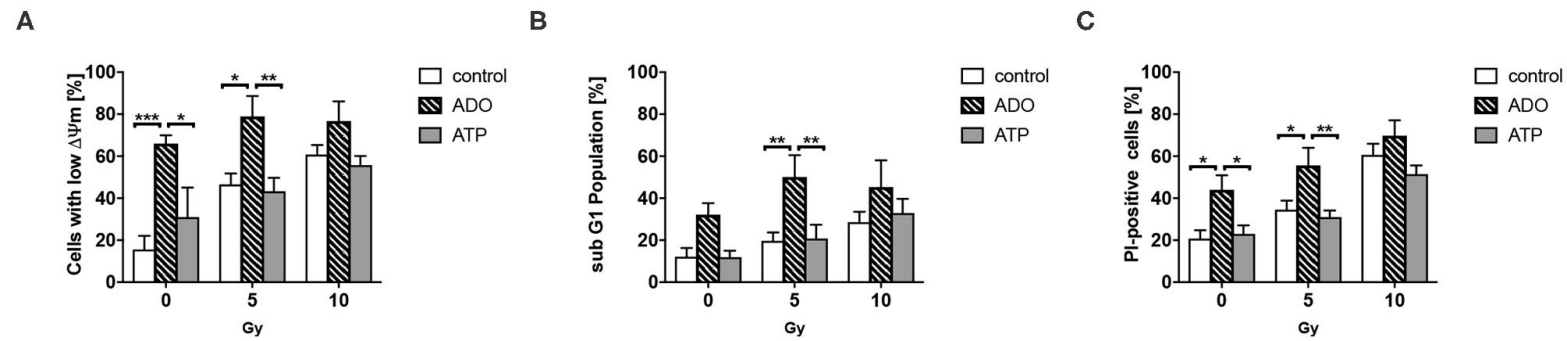
Effects of extracellular ATP and Ado on LLC1 tumor cells *in vitro*. LLC1 cells were incubated with ATP (2 mg/mL) or Ado (1 mg/mL) 1 h prior to 0-, 5-, or 10-Gy irradiation. Forty-eight hours later, cells were stained with TMRE or PI and further analyzed with flow cytometry to investigate the **(A)** mitochondrial potential (ΔΨm), **(B)** the sub-G1 fraction (apoptosis), and **(C)** PI-positive cells (cell death). Data show means ± SEM; *P ≤ 0.05, **P ≤ 0.01, ***P ≤ 0.001 by two-way ANOVA followed by *post hoc* Tukey test.

ATP and Ado have been described to impact the survival of cancer cells (74–76). In complementary *in vitro* investigations, we therefore aimed to explore if high ATP or Ado levels would impact cancer cell survival. Therefore, we determined the effects of exogenously added extracellular ATP and Ado in combination with irradiation on short-term cell survival (Figure 3). In untreated cells, irradiation induced a dose-dependent increase in cells with low mitochondrial potential (Figure 3A), in the sub-G1 population (Figure 3B), and in PI-positive cells (Figure 3C) 48 h after irradiation, which is indicative for the induction of apoptosis and total cell death, respectively. Similar results were obtained when ATP was added to the culture medium 1 h before irradiation up to a concentration of 1 mg/mL. Despite a trend to single-drug toxicity of exogenously added ATP on LLC1 cells, high ATP levels in the culture medium did not alter the toxic effects of irradiation with 5 or 10 Gy in these cells (Figures 3A–C). In contrast, very high extracellular Ado concentrations (2 mg/mL) were highly toxic to the LLC1 cells, as indicated by the significant increase in the fraction of LLC1 cells with a low mitochondrial potential, in the sub-G1 population, and of PI-positive cells (Figures 3A–C). Yet, neither a further increase in toxicity nor a protective effect of high Ado levels in the culture medium was observed in LLC1 cells in combination with irradiation (Figures 3A–C).

### CD39 Deficiency in the Tumor Host Did Not Significantly Alter Radiation-Induced Tumor Immune Responses

**Figure 4:**
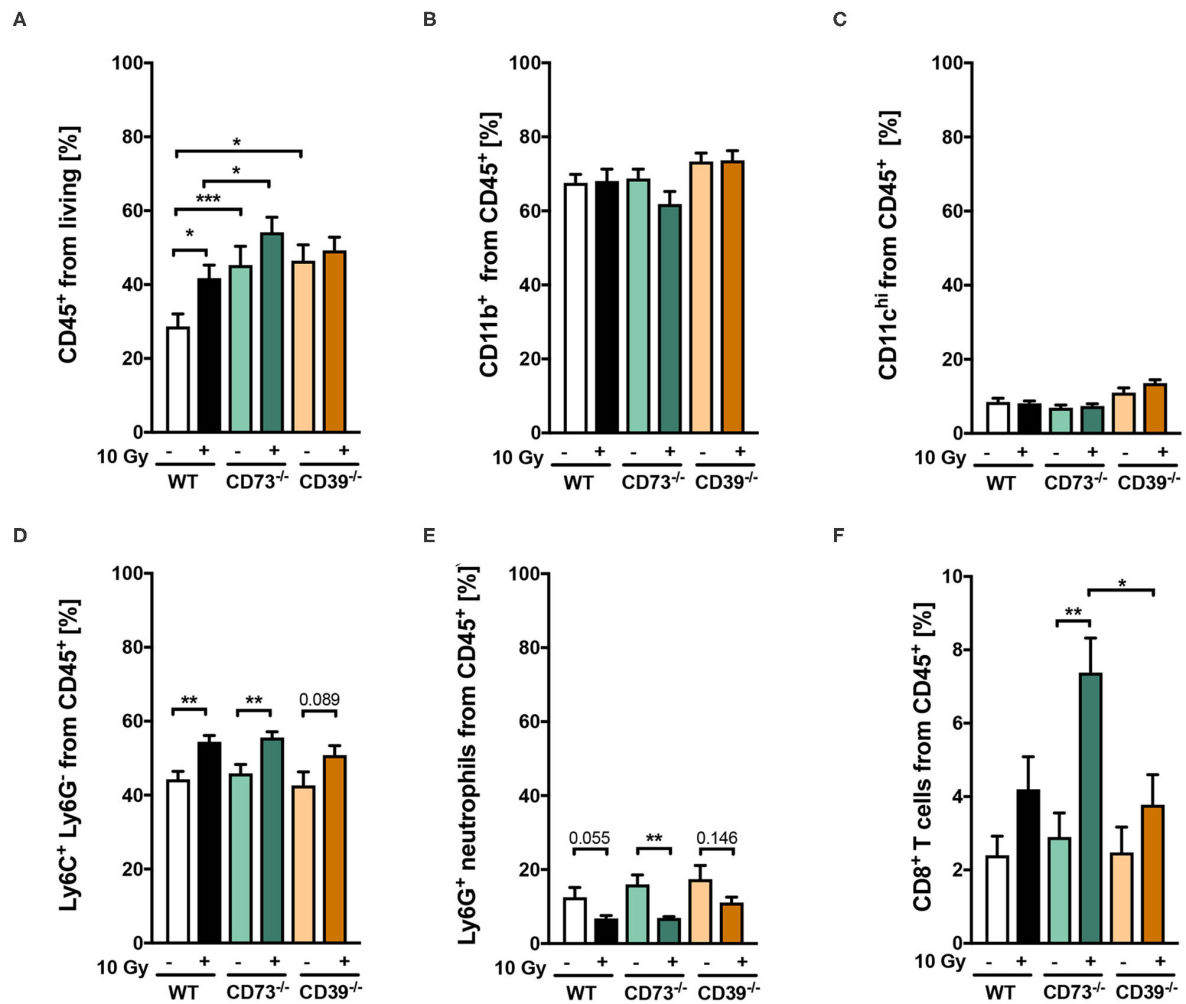
**(A-F)** Host CD39 deficiency causes only subtle changes in radiation-induced tumor immune cell infiltration. LLC1 tumor cells (0.5 × 10^6^ cells each) were subcutaneously transplanted onto the hindlimb of C57BL/6 wild-type (white/black) CD73-knockout (CD73^−/−^; green, triangles) and CD39-knockout mice (CD39^−/−^; orange circles) mice. Hind-leg single-dose irradiation with 0 or 10 Gy was conducted at the timepoint of tumor manifestation. Three days after irradiation, tumors were dissected, and single-cell suspensions were generated. Tumor-infiltrating leukocytes were analyzed via flow cytometry. Shown in bar diagrams are the percentages of diverse immune cell populations. Shown are means ± SEM, *P ≤ 0.05, **P ≤ 0.01, ***P ≤ 0.001, analyzed by one-way ANOVA followed by a *post hoc* Tukey test, *n* = (13/14) (C57BL/6), (9/9) (CD73^−/−^), (10/9) (CD39^−/−^).

As ATP and Ado are well-known potent immunomodulators, we next investigated a potential contribution of differences in infiltrating immune cells within LLC1 tumors to the observed effects by quantification and phenotypic characterization of immune cell subsets using flow cytometry. To study an initial irradiation-induced immune infiltration, tumors were harvested 3 days after irradiation with 0 Gy (sham controls) or 10 Gy and subjected to immune cell analysis (Figure 4). Irradiation of LLC1 tumors grown on WT mice induced a significant increase in the percentage of CD45^+^ leukocytes in the tumor tissue at 3 days after irradiation (Figure 4A). In contrast to WT mice, unirradiated LLC1 tumors grown on CD39^−/−^ or CD73^−/−^ mice already had higher percentages of CD45^+^ leukocytes. While exposure to a single dose with 10 Gy did not significantly increase the number of CD45^+^ leukocytes in LLC1 tumors grown on CD39^−/−^ mice, there was a significantly higher leukocyte number detected in irradiated tumors growing on CD73^−/−^ mice compared to tumors grown on WT mice (Figure 4A).

Furthermore, no significant differences were detected for different immune cell subsets, in unirradiated LLC1 tumors growing on either WT, CD39^−/−^, or CD73^−/−^ mice, including neutrophils, monocytic cells, dendritic cells, and cytotoxic T lymphocytes (Figures 4B–F). Moreover, exposure to irradiation did not increase the infiltration either of CD11b^+^ myeloid cells (Figure 4B) or of CD11c^hi^ dendritic cells (Figure 4C) into LLC1 tumors at day 3 after irradiation in the three different mouse strains. In contrast, a significant increase in the percentage of infiltrating Ly6G^−^ Ly6C^+^ monocytic cells and a significant decrease in the percentage of Ly6G^+^ Ly6C^−^ neutrophils among tumor-infiltrating CD45^+^ immune cells were detected in irradiated tumors from WT and CD73^−/−^ mice (Figures 4D,E). Instead, radiation-induced changes in the myeloid compartment of tumors grown on CD39^−/−^ mice were not statistically significant (Figures 4D,E). Interestingly, exposure of LLC1 tumors to a single dose of 10 Gy induced a significantly increased percentage of CD8^+^ cytotoxic T cells in tumors grown on CD73^−/−^ mice (Figure 4F), whereas no difference in the percentage of cytotoxic T cells was observed in irradiated tumors grown on WT and CD39^−/−^ mice (Figure 4F).

Taken together, irradiation of LLC1 tumors with a single dose of 10 Gy induced a significant increase in immune cell infiltration when grown on WT (monocytic cells) and CD73^−/−^ mice (monocytic cells, CD8^+^ T cells) but not when grown on CD39^−/−^ mice. However, because the basal infiltration of CD45^+^ immune cells was significantly higher when LLC1 tumors grew on CD39^−/−^ mice when compared to WT mice, we assumed that the subtle differences in the observed immunological parameters upon irradiation might have potential tumor promoting effects, because infiltrating myeloid cells are the majority of immune cells.

To elucidate if the non-canonical adenosinergic pathway might have an impact on a radiation-induced tumor response and immunomodulation, we analyzed the expression of CD38 and CD203a/PC-1 in LLC1 tumors grown on WT, CD39^−/−^, and CD73^−/−^ mice. As shown in Supplementary Figure 2A, quantitative reverse transcriptase (qRT)–PCR analysis revealed a CD38 mRNA expression in LLC1 cells that further increased after irradiation. However, we could not detect any CD203a mRNA expression in LLC1 cells before and after irradiation. We further performed IHC staining from paraffin-embedded tumor tissue sections from LLC1 hind-leg tumors grown on WT mice for CD38 and CD203a protein expression. Surprisingly as shown in Supplementary Figure 2B, LLC1 tumors itself did not express CD38 or CD203a protein, nor were there any obvious CD38/CD203a positive immune or stroma cells. Moreover, irradiation did not alter the expression, nor did the different tumor hosts (WT, CD39^−/−^, and CD73^−/−^) have an effect on the expression of both receptors (data not shown). Our anti-CD38 staining from the liver tissue as positive control revealed the specificity of this antibody (Supplementary Figure 2B, right panel). Identical results were obtained from LLC1 tumors grown on CD39^−/−^ and CD73^−/−^ mice (data not shown). Nevertheless, because our data clearly demonstrate that CD203a is not expressed on gene and protein level, we hypothesize that independent of a potential CD38 expression, the adenosinergic non-canonical pathway cannot function in the tumor to produce AMP to fuel CD73.

### Host CD39 Deficiency Caused a Reduced Regressive Phenotype of Irradiated Tumors

**Figure 5:**
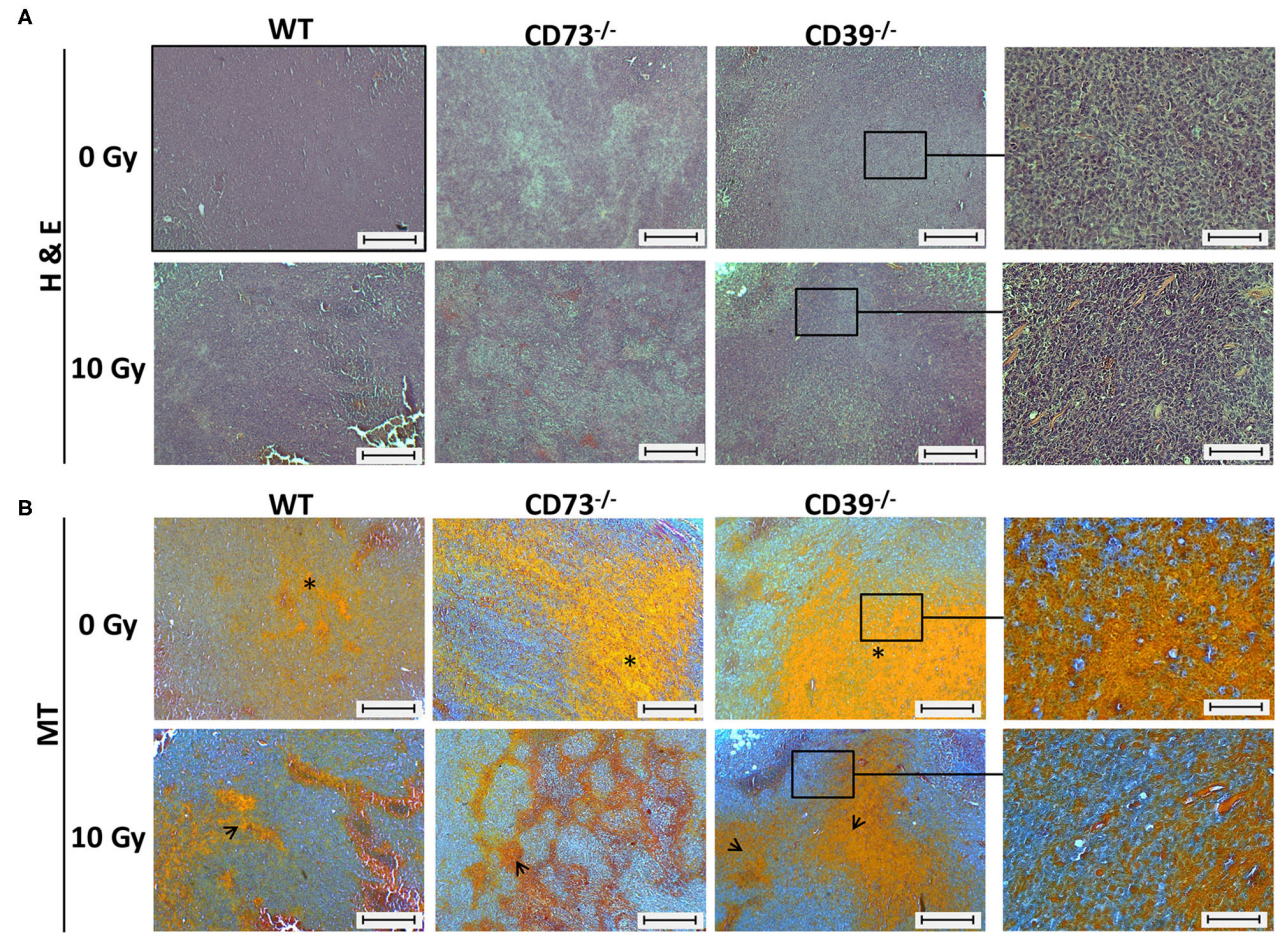
Host CD39 deficiency causes a reduced regressive phenotype of irradiated tumors. LC1 tumor cells (0.5 × 10^6^ cells each) were subcutaneously transplanted onto the hindlimb of C57BL/6 wild-type and CD39- and CD73-deficient [knockout (^−/−^)] mice. Hind-leg single-dose irradiation with 0 or 10 Gy was conducted at the timepoint of tumor manifestation. Mice were sacrificed at the timepoint of maximal tumor volume (1,000 mm^3^). **(A)** Hematoxylin-eosin (H&E) or **(B)** Masson Goldner trichrome (MT) staining of paraffin-embedded LLC1 tumor tissue, scale bar 100 μm. Magnified pictures (400-fold) highlight special structures. *n* = 15/17 (C57BL/6), 17/13 (CD39^−/−^), 7/13 (CD73^−/−^). Asterisks and open arrows indicate areas of a higher degree of epithelial structures (orange colored).

Because CD39 is also important to the function of stromal cells, e.g., fibroblasts, mesenchymal stem cells, and vascular cells, we aimed to explore a potential contribution of an altered function of stromal cells in CD39^−/−^ mice to the observed differences in the radiation response of tumors grown CD39^−/−^ mice vs. WT and CD73^−/−^ mice. Therefore, we performed a detailed histological analysis of the respective tumor sections using H&E and MT staining (Figure 5).

All control (0 Gy) tumors isolated from the different mouse strains were generally characterized by a densely packed epithelial cell structure, and no obvious histological differences could be detected between tumors grown on WT, CD73^−/−^, or CD39^−/−^ mice (Figure 5A, upper panels). As expected, exposure to ionizing radiation (IR) induced regressive tissue alterations, as visualized by the reduced packaging of tumor cells, increased areas of necrosis and of connective tissue structures, and an altered morphology of the cell nuclei (violet staining), respectively (Figure 5A, lower panels). Of note, while tumors from WT mice and CD73^−/−^ mice seemed to be similarly affected following IR, less regression and thus more remaining tumor cells became visible in tumors grown on CD39^−/−^ background, indicating a reduced degree of tissue damage (Figure 5A, right panel). Additional Masson trichrome (MT) staining confirmed a higher degree of epithelial structures (orange colored) in tumors of CD39^−/−^ mice, which might be indicative for an altered tumor cell proliferation state (Figure 5B).

**Figure 6:**
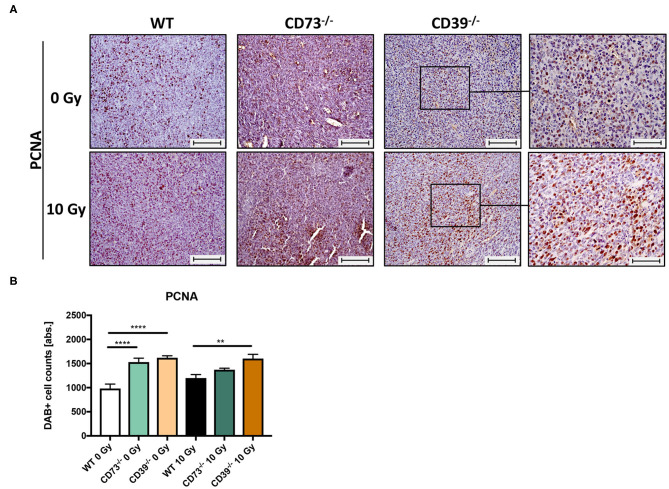
LLC1 tumor grown on CD39^−/−^ mice are not impaired in their proliferative activity after irradiation. LLC1 tumor cells (0.5 × 10^6^ cells each) were subcutaneously transplanted onto the hindlimb of C57BL/6 wild-type and CD39- and CD73-deficient [knockout (^−/−^)] mice. Hind-leg single-dose irradiation with 0 or 10 Gy was conducted at the timepoint of tumor manifestation. Mice were sacrificed at the timepoint of maximal tumor volume (1,000 mm^3^). IHC staining of proliferation cell nuclear antigen (PCNA) of paraffin-embedded LLC1 tumor tissue, magnification 200-fold, scale bar 100 μm **(A)**. Shown in bar diagrams are the absolute numbers of DAB^+^ cell counts per tissue section **(B)**. Magnified pictures (400-fold) highlight special structures. Shown are means ± SEM, **P ≤ 0.01, ****P ≤ 0.0001, analyzed by one-way ANOVA followed by a *post hoc* Tukey test *n* = 15/17 (C57BL/6), *n* = 17/13 (CD39^−/−^), *n* = 7/13 (CD73^−/−^).

So far, our data revealed that LLC1 grown on a CD39-deficient mouse grew faster and were less sensitive to the growth-limiting effects of RT. For further histological evaluation of the tumor tissue sections, and to specify if LLC1 tumors grown on CD39^−/−^ mice have altered levels viable tumor cells and necrosis after irradiation, we investigated the proliferative activity in the tumors by immunohistological staining of the PCNA (Figure 6). In the respective unirradiated conditions (Figure 6A, upper panel), proliferating cells were found in all tumors independent of the mouse strain. Even in irradiated tumors (10–14 days after irradiation), strong PCNA immunoreactivities were detected in tumors isolated from all three mouse strains (Figure 6A, lower panel). Quantification of the PCNA^+^ tumor cells using an automatic DAB-positive (brown) cell counting procedure (semiquantitative analysis) revealed that in unirradiated tumors grown on CD73^−/−^ and CD39^−/−^ mice, significantly more PCNA^+^ cells were present than in tumors grown on WT mice (Figure 6B). Even more important, tumors grown on CD39^−/−^ mice had significantly more PCNA^+^ cells than tumors grown on WT mice and showed no impairment in their proliferative activity after a single high-dose irradiation with 10 Gy. In line with the results obtained from the histological analyses, our findings indicate that irradiation causes less tissue damage and necrosis in tumors grown on CD39^−/−^ mice when compared to tumors grown on WT or CD73-deficient mice. Thus, the loss of CD39 in the tumor host did not result in more pronounced tissue damage and inflammation upon a single high-dose irradiation with 10 Gy but instead in a more pronounced proliferation.

### Enhanced Growth and Resistance to Radiation-Induced Growth Delay of LLC1 Tumors Grown on CD39-Deficient Hosts Are Linked to Alterations in the Tumor Endothelial Compartment

**Figure 7:**
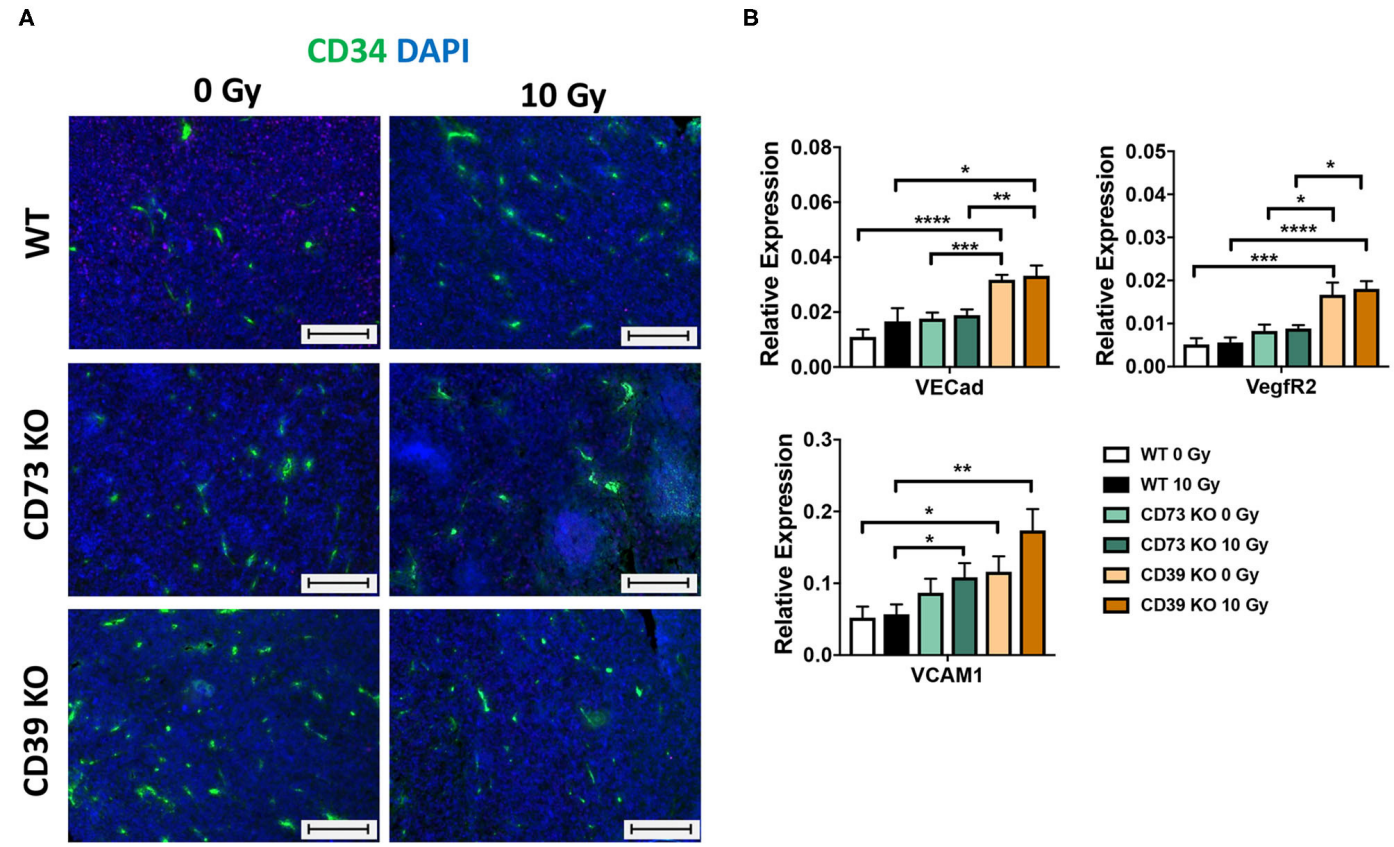
LLC1 tumor grown on CD39^−/−^ mice show an altered microvasculature. LLC1 tumor cells (0.5 × 10^6^ cells each) were subcutaneously transplanted onto the hindlimb of C57BL/6 wild-type and CD39- and CD73-deficient [knockout (^−/−^)] mice. Hind-leg single-dose irradiation with 0 or 10 Gy was conducted at the timepoint of tumor manifestation. Mice were sacrificed at the timepoint of maximal tumor volume (1,000 mm^3^). **(A)** Immune fluorescent staining of CD34 (green) and DAPI (blue) of paraffin-embedded LLC1 tumor tissue, magnification 200-fold, scale bar 100 μm. 15/17 (C57BL/6), *n* = 17/13 (CD39^−/−^), *n* = 7/13 (CD73^−/−^). **(B)** RT qPCR analysis to reveal the relative expression of VE Cad, VegfR2, and VCAM1 to actin. The relative expression is shown in bar diagrams. Shown are means ± SEM, *P ≤ 0.05, **P ≤ 0.01, ***P ≤ 0.001, ****P ≤ 0.0001, analyzed by one-way ANOVA followed by a *post hoc* Tukey test *n* = 12/12 (C57BL/6), *n* = 12/12 (CD39^−/−^), *n* = 12/12 (CD73^−/−^).

Although not being directly obvious from the histological evaluation, we had the impression that the vasculature was altered in tumors growing on CD39-deficient hosts. We therefore performed immunofluorescence staining of tumor sections using the angiogenic endothelial cell marker CD34 (Figure 7A). As shown in Figure 7A (left panels), already unirradiated tumors grown on CD39^−/−^ mice had more CD34^+^ immunoreactive structures and increased microvascular densities as compared to tumors grown on WT or CD73^−/−^ mice. Importantly, differences in microvascular density were still present after exposure of the tumors to a single high-dose irradiation with 10 Gy (Figure 7A, right panels). To further characterize the assumed differences in the vascular compartment, a qRT-PCR was performed using vascular endothelial cadherin (VE-Cad), the major angiogenic growth factor receptor VegfR2 and vascular cell adhesion protein 1 (VCAM1) as markers for tumor vascularization (Figure 7B). Indeed, tumors grown on CD39^−/−^ mice displayed significantly enhanced expression levels of VE-Cad, VegfR2, and VCAM1 already under control conditions (0 Gy), and these differences persisted also upon irradiation as indicated by significantly increased levels of all three vascular markers, in tumors grown on CD39^−/−^ mice compared to tumors grown on WT controls (Figure 7B). In contrast to CD39^−/−^ mice, tumors grown on CD73^−/−^ mice had similar expression level as tumors grown on WT mice. We could only detect a significantly increased expression of VCAM1 in irradiated tumors grown on CD73^−/−^ mice compared to WT animals.

These data strongly suggest that loss of CD39 in the tumor host and the resulting changes in the TME enhance the proliferation capacity and growth of LLC1 tumors, and this was associated with alterations in the vascular compartment particularly with an increased microvascular density. The reduced sensitivity of CD39-deficient microvessels and the cytotoxic effects of RT observed in the present study particularly in CD39–/– mice may contribute to the reduced radiation-induced tumor growth delay of LLC1 tumors and thus impact on radiation resistance.

### Genetic Deficiency of CD39 Exacerbates Radiation-Induced Lung Fibrosis

**Figure 8:**
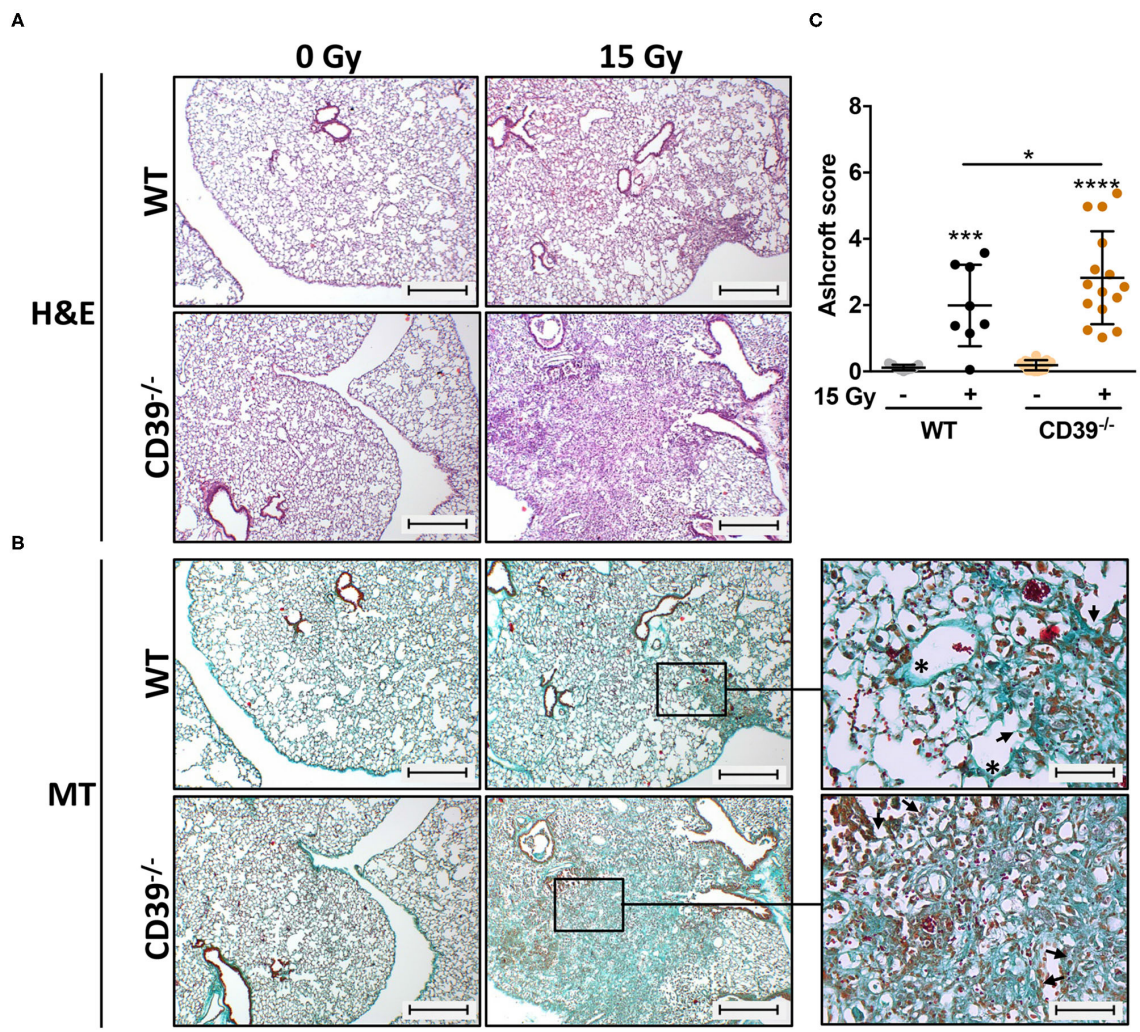
CD39^−/−^ mice develop enhanced pulmonary fibrosis 25 weeks after irradiation. C57BL/6 (WT) and CD39^−/−^ mice received 0- or 15-Gy whole-thorax irradiation (WTI) and were sacrificed at 25 weeks after irradiation. **(A)** Hematoxylin-eosin (H&E) or **(B)** Masson Goldner trichrome (MT) stained lung sections at 25 weeks after irradiation. Lung sections were evaluated for fibrosis by using 10 non-overlapping randomized pictures (scale bar = 100 μm) per slide. Ashcroft scoring was done by three independent observers on slides blinded to the genotype and treatment condition. Magnified pictures (400-fold) highlight special structures. Asterisks emphasize thickening of alveolar wall structures and arrowheads fibrotic regions. **(C)** Quantification of fibrosis in WT (*n* ≥ 7) and CD73^−/−^ (*n* = 7) mice, horizontal lines represent mean values. *P ≤ 0.05, ***P ≤ 0.001, **** *P* ≤ 0.0001 by one-way ANOVA followed by *post hoc* Newman–Keuls test, *n* = 9/9/15/15.

A major challenge for novel strategies to improve RT outcome is to enhance tumor cell killing while limiting the risk for radiation-induced adverse effects in highly radiosensitive normal tissues such as the lung. The suggested dual role of ATP/CD39 in cancer and fibrosis and the adverse effects of CD39 deficiency in the tumor host on growth and radiation-response of LLC1 lung tumors prompted us to investigate in addition if genetic deficiency of CD39 would also alter radiation-induced adverse effects in normal lung tissue. Therefore, we examined the effects of a single high-dose WTI (15 Gy) on fibrosis development in CD39^−/−^ mice compared to WT mice in our murine model of RT-induced lung disease (49). A detailed histological analysis of lungs isolated from CD39^−/−^ mice and respective WT controls revealed a prominent thickening of alveolar septa, increased extracellular matrix deposition, and multiple fibrotic foci, characteristic for fibrosis development in irradiated WT mice as of 25 weeks after WTI (Figures 8A,B). Of note, a more severe fibrosis phenotype was prominent in lung sections of irradiated CD39^−/−^ mice, as demonstrated by a further increase in extracellular matrix deposition and more severe fibrotic lesions yielding a significantly higher Ashcroft score (Figure 8C).

Because the observed results might not only be evoked by the canonical CD39/CD73/Ado pathway, we again addressed the non-canonical adenosinergic pathway in respective lung sections by using immunohistochemistry in combination with CD38 and CD203a/PC-1 antibodies. As shown in Supplementary Figure 3, our data revealed that both receptors CD38 and CD203a were not expressed in the alveolar and bronchial lung regions from untreated WT mice (Supplementary Figure 3, left panel). Even fibrotic alveolar and bronchial lung regions of irradiated WT mice did not express these receptors (Supplementary Figure 3, middle panels). CD38 and also CD203a expression was observed in only few numbers of small infiltrating immune cells, with a myeloid morphology, in the irradiated lungs (Supplementary Figure 3, magnification, right panel). The observed findings were true for WT, CD39^−/−^, and CD73^−/−^ mice, with no difference in the expression between all strains. Our results reveal that CD38/CD203a/CD73 ectoenzymatic signaling on infiltrating immune cells (monocytes/macrophages) might also impact irradiation-induced pneumopathy. However, expression of non-canonical ectoenzymes was similar in lungs from WT, CD39^−/−^, and CD73^−/−^ mice; thus, we conclude that the observed exacerbated fibrosis development in CD39^−/−^ mice is independent from the non-canonical pathway.

**Figure 9:**
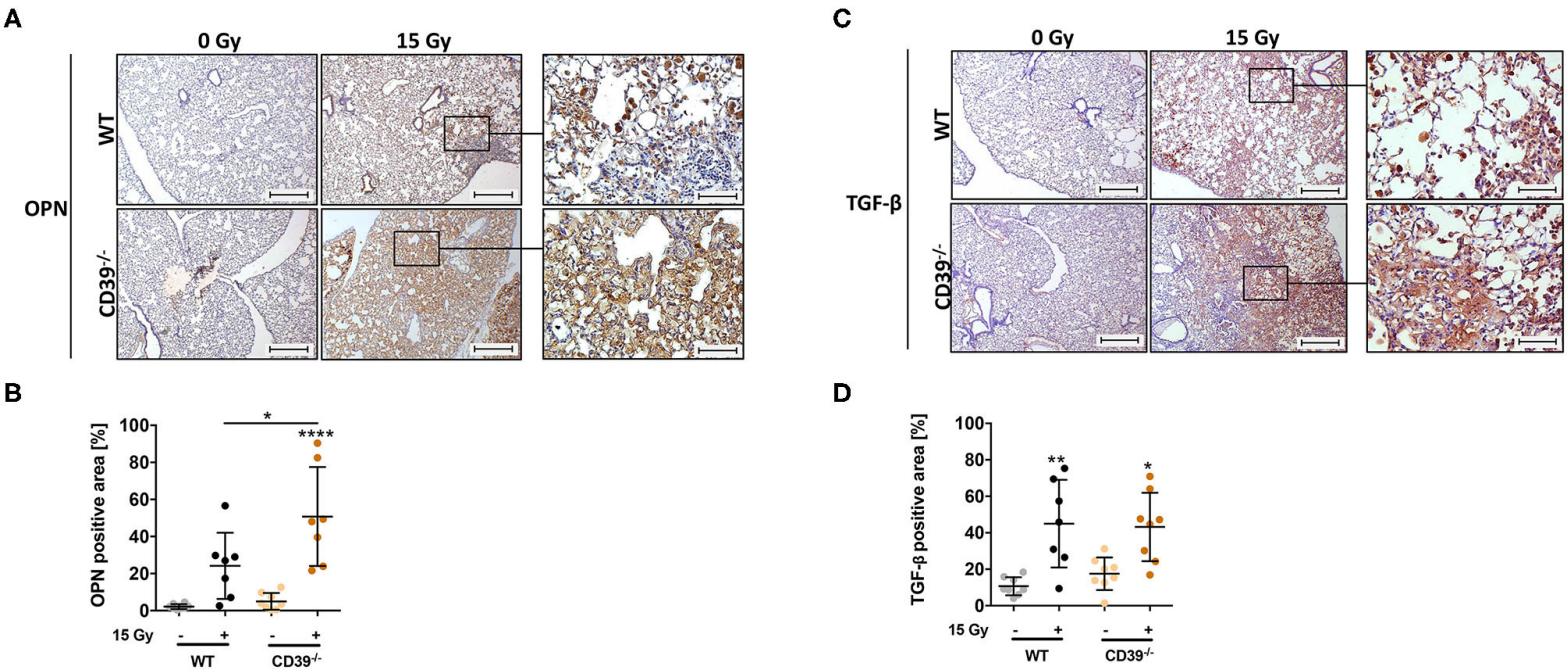
A CD39 deficiency leads to an increased osteopontin expression and TGF-β accumulation after irradiation. C57BL/6 (WT) and CD39^−/−^ mice received 0- or 15-Gy whole-thorax irradiation (WTI) and were sacrificed at 25 weeks after irradiation. Immunohistochemical staining of paraffin-embedded lung sections with a primary antibody for **(A,B)** osteopontin (OPN) and **(C,D)** transforming growth factor β (TGF-β). Shown in (**A,C** right panels) are representative overview pictures of each indicated group (scale bar = 100 μm, magnified pictures 400-fold). **(B,D)** Using the Software Orbit Image Analysis version 2.65, positive areas for OPN and TGF-β were analyzed and are shown in percentage of the whole lung section. *P ≤ 0.05, **P ≤ 0.01, ****P ≤ 0.0001 by one-way ANOVA followed by *post hoc* Newman–Keuls test, *n* = 8/7/8/8.

Our previous work revealed that progressive activation of CD73 and chronic accumulation of Ado participated in shaping a profibrotic cross-talk between damaged resident cells, infiltrating immune cells, and other fibrosis mediators such as OPN, hyaluronic acid, and TGF-β (49, 50). To corroborate the induction of fibrosis and gain insight into potential underlying mechanisms, we additionally analyzed the expression levels of the profibrotic markers OPN and TGF-β, both known to be associated with fibrosis development, via immunohistochemistry (49, 77, 78). In line with the above observations, WTI induced clearly higher levels of OPN in the lungs of CD39^−/−^ mice as compared to respective WT controls at 25 weeks after irradiation, particularly in the fibrotic areas (Figures 9A,B). Yet TGF-β was expressed to a similar extent in the lungs of both mouse strains (Figures 9C,D). Moreover, the time-dependent changes in infiltration of CD45^+^ leukocytes were rather similar in both mouse strains except for slightly more pronounced decrease in the fraction of CD45^+^ leukocytes in the lung tissue at 1 and 24 weeks after irradiation (data not shown).

Thus, genetic loss of CD39 did not only adversely affect the tumor response to RT but even correlated with an increased normal lung toxicity upon IR as revealed by the significantly enhanced progression of radiation-induced lung fibrosis.

## Discussion

Beyond CD73 and Ado, CD39 emerges as an attractive therapeutic target for cancer therapy. So far, the role of the purinergic pathway, particularly CD39-dependent signaling, in radiation responses of normal and tumor tissues remains largely unknown. Here, we demonstrated that host CD39 impacts both tumor growth and tumor and normal tissue responses to IR; murine LLC1 lung tumors grew faster on mice with genetic deficiency of CD39 and were more resistant to tumor growth delay induced by single high-dose irradiations, whereas genetic deficiency of CD73 in the tumor host did not significantly alter tumor growth and the response to RT. Radiation-induced lung fibrosis, however, was more severe in CD39^−/−^ as compared to WT mice. The assumed defect of CD39-deficient host cells in degrading extracellular ATP did not foster tumor cell damage or increased immune cell infiltration upon irradiation, but increased tumor cell proliferation and survival. The increased tumor cell proliferation observed in LLC1 tumors of CD39-deficient mice (either or without RT treatment) was associated with increased angiogenesis. Thus, host CD39 supports the antineoplastic effects of RT in the murine LLC1 lung cancer model, while limiting radiation-induced fibrosis in respective mice.

### The Role of CD39 Within Lung Tumors

In more detail, we used syngeneic LLC1 lung tumors xenografts grown on CD39- and CD73-deficient mice as compared to respective WT controls in combination with a single high-dose irradiation. LLC1 lung cancer cells do express neither CD39 nor CD73, thereby excluding potential confounding effects by tumor-associated CD39/CD73 signaling. Interestingly, LLC1 tumors grew faster on CD39^−/−^ mice, whereas loss of CD73 had no significant effect on LLC1 tumor growth. Even more important, irradiation with 10 Gy was not sufficient to induce similar antitumor effects in tumors grown on CD39^−/−^ mice as compared to WT mice, while radiation-induced growth retardation of tumors grown in CD73^−/−^ mice was similar. Our initial idea was that genetic deficiency of CD39 in mice will inhibit degradation of extracellular ATP released into the microenvironment in response to radiation-induced tissue damage and that the resulting increase in ATP and reduced production of Ado might lead to increased activation of inflammatory responses. Regardless of the fact that unirradiated tumors grown on CD39^−/−^ and CD73^−/−^ mice already had higher percentages of tumor-infiltrating leukocytes, our flow cytometry data revealed that exposure to a single high-dose irradiation did not significantly increase the percentage of leukocytes and respective subsets infiltrating LLC1 tumors grown on CD39^−/−^ mice after irradiation. Despite the fact that we found infiltrating leukocytes in all tumors (WT, CD73^−/−^, and CD39^−/−^), the poor overall immune cell infiltration observed at this time point corroborates the reported poor immunogenicity of LLC1 tumors (79).

Instead, LLC1 tumors grown on CD73^−/−^ mice showed a higher percentage of cytotoxic T cells in the tumor tissue at day 3 after irradiation than LLC1 tumors growing on WT and CD39^−/−^ mice. This is reminiscent of a recent study describing that a CD73-targeted therapy increased CD8^+^ T-cell infiltration in a murine mammary carcinoma cell line TSA tumor hind-leg model only in combination with additional single high-dose tumor irradiation (20 Gy) (80). Yet, in our hands, the observed increased CD8^+^ T-cell infiltration did not result in a significant effect on tumor growth delay. Thus, although deficiency of CD73 in the tumor host may reduce Ado levels in the tumor micromilieu and thereby facilitate radiation-induced antitumor immune responses as observed by others (24), the effects resulting from a single high-dose irradiation were not strong enough to induce net effects on LLC1 tumor growth retardation. Further work is required to define time-dependent changes in the micromilieu of irradiated tumors induced in response to IR in the different genetic backgrounds. Moreover, similar investigations should be performed using fractionated radiation schedules, tumor cells with higher immunogenicity, and CD39/CD73-positive tumor cells. In fact, CD73 overexpression has been described to be higher in tumors than in surrounding normal tissues and also to be tumor-specific (24).

In addition to its role in the regulation of inflammatory processes ATP has been described to modulate proliferation and death resistance of cancer cells. Human lung cancer cells were shown to use macropinocytosis or clathrin- and caveolae-mediated endocytotic processes to take up extracellular ATP and fuel extra energy for tumor growth, survival, and drug resistance (81). Moreover, while ATP inhibited proliferation or induced cell death via P2X7R in human cervix and breast cancer cells as well as in murine melanoma and colon cancer cells (76, 82–85), high levels of extracellular ATP promoted survival of A549 and H23 lung cancer cells as compared to normal cells (86); here, the authors attributed the differential effects on tumor and normal tissue cells to the decreased expression of P2X7R and an enhanced Bcl-2/Bax ratio in the cancer cells (86). Because our *in vitro* investigations revealed that LLC1 cells do not express P2X7R and did not undergo increased cell death in response to extracellular ATP alone or in combination with RT, we speculate that similar processes might contribute to the observed growth and resistance promoting effects of CD39 deficiency in the tumor host. Besides P2 receptor signaling, nucleotides can also act through intracellular uptake and compartmentalization with impact on cancer cell survival, proliferation, and metabolic function (75). In fact, with increasing concentration, extracellular Ado can enter the cell via equilibrative nucleoside transporters (ENTs) and subsequently induce cell death (87, 88). Herein, ATP degradation to Ado and subsequent uptake and conversion of Ado to AMP by ENT and Ado kinase, respectively, were shown to be the main drivers of growth inhibition and toxicity mediated by extracellular ATP (76, 89). High extracellular Ado levels could activate mitochondrial apoptosis through deregulation of the Bcl2 rheostat by decreasing antiapoptotic Bcl2 while increasing expression of proapoptotic Bax and Bak (90). Degradation of ATP to Ado induced growth inhibition and proapoptotic effects via activation of caspase-3 (91). Finally, depending on the concentration, AMP turned out to strongly inhibit cancer cell proliferation *in vitro* even more effective than Ado and ATP (92).

Ado signaling via the four ADORA receptors could alternatively impact tumor growth and survival in the LLC1 cells, because these cells express the ADORA receptors. The effect of Ado via both AdoRA1 and A3 has been described as proapoptotic in several cancer types, e.g., liver, lung, and colon (91, 93–96). The AdoRA1, for example, had an antiproliferative effect and promoted the differentiation of cancer stem cells, thereby inducing an increased sensitivity of these cells to chemotherapeutic drugs (97). Furthermore, treatment with an agonist of AdoRA3 reduced the number of living colon cancer cells (98). Thus, alterations in extracellular nucleotides (ATP < -> Ado) due to the loss of host CD39 in our LLC1 tumor model could potentially affect tumor growth and the radiation response via ADORA signaling as well as through intracellular uptake and metabolic processing, independent of an immune response and P2 receptor signaling. Host CD39 expression seems to be important for maintaining a radiation-induced antitumor effect in CD39/CD73-negative LLC1 presumably by affecting the composition of extracellular nucleotides in the TME. Further work is needed to elucidate the effects of extracellular nucleotide uptake and subsequent intracellular nucleotide metabolism in LLC1 tumor development.

Because alternative nucleotide signaling might also contribute to our observed findings, we analyzed the role of the non-canonical adenosinergic CD38/CD203a/CD73 pathway. Although we could detect CD38 mRNA expression in LLC1 tumors, there was a lack of CD38 cell surface protein. By analyzing a panel of human lung cancer cell lines, Bu et al. (99) already described that most cell lines investigated had high copy numbers of CD38 mRNA, but not all cell lines expressed the respective protein. Moreover, we showed here that there was no CD203a mRNA and protein expression needed to produce Ado and fuel CD73. Therefore, we can exclude that non-canonical adenosinergic signaling in the LLC1 tumor tissue contributes to the observed tumoral behavior reported here. In addition to catalyzing the production of Ado, CD38 may promote tumor development by inducing other tumor-supporting processes, e.g., angiogenesis in the TME, thus exerting functions beyond the CD38/CD203a/CD73 signaling (100). We observed here that tumors grown on CD39-deficient background were characterized by an increased neovascularization and that this phenotype was even maintained after RT. Radiation usually induces phenotypic changes of the tumor vasculature (e.g., apoptosis or senescence), as well as a wide range of environmental changes, which in turn govern recruitment of immune cells (101–104). Herein, angiogenic and thus less mature blood vessels are characterized by an increased radiosensitivity (61, 73, 105). We speculate that the sustained angiogenesis observed in the context of CD39 deficiency might result from increased proangiogenic signaling induced by higher extracellular ATP levels due to the failure of extracellular ATP degradation and the increased ATP release from RT-damaged cells. At the same time, extracellular ATP has been reported to mediate antiapoptotic effects in endothelial cells, either via P2 or Ado receptors (106). Likewise, CD39-deficient endothelial cells might be less sensitive to the cytotoxic effects of RT, thereby contributing to the reduced sensitivity of LLC1 tumors to radiation. ATP was already shown being able to activate the VEGF receptor in the absence of VEGF, and the P2YR–VEGFR2 interaction and the resulting signal transduction are critical determinants of vascular homoeostasis and tumor-mediated angiogenesis (107). Moreover, CD38-dependent signaling can impact on (tumor) angiogenesis (55, 108). Herein, CD38 turned out to be an important regulator for the intrinsic expression of key angiogenic factors (100, 109, 110). Although non-canonical purinergic signaling seemed not to impact on non-canonical ADO generation because of the lack of CD203a expressions as reported here, increased tumor angiogenesis together with the well-known abnormal phenotype of the tumor vasculature may lead to factors contributing to radiation resistance (increased vascular permeability, vessel instability, increased interstitial fluid pressure, non-directed blood flow, endothelial anergy) (104, 111, 112).

### The Role of CD39 Within Normal Lung Toxicity

Concerning normal tissue toxicity, we showed here that genetic loss of CD39 exacerbates radiation-induced lung disease. Since our earlier work revealed a pathogenic role of the CD73/Ado-dependent arm of purinergic signaling in radiation-induced lung fibrosis (49, 50), genetic loss of CD39 with assumed activation of the ATP-dependent proinflammatory arm of purinergic signaling and genetic loss of CD73 with assumed activation of the Ado-dependent anti-inflammatory, repair-promoting arm of purinergic signaling result in opposing outcomes in radiation-induced lung fibrosis. A pathology-promoting effect of low CD39 expression or high pulmonary ATP levels were already known for several lung pathologies, including pulmonary fibrosis (113–115). High extracellular ATP levels, for example, have been observed in the bronchoalveolar lavage fluid of patients with idiopathic pulmonary fibrosis, as well as in a murine model of bleomycin-induced lung fibrosis (116). In our hands, the more severe lung fibrosis observed in irradiated lungs from CD39^−/−^ mice was associated with enhanced levels of the multifunctional and proinflammatory protein OPN in the irradiated lungs CD39^−/−^ mice compared to WT mice. Based on the observation that extracellular ATP can induce OPN expression in response to mechanical stress at least *in vitro* (117), we speculate that genetic deficiency of CD39 may cause a disease-promoting increase in extracellular ATP concentrations in irradiated lungs, presumably in response to the initial radiation damage in irradiated murine lungs. CD39-dependent vascular effects may also play a major role in regulating severity of radiation-induced fibrosis. Increasing evidence suggests that vascular remodeling contributes to the pathogenesis of pulmonary fibrosis (66, 73, 118, 119). Of note, loss of CD39 induced vascular remodeling of pulmonary arteries and contributed to vascular dysfunction and arterial hypertension in patients suffering from idiopathic pulmonary arterial hypertension (120, 121). However, further work is needed to elucidate the contribution of vascular changes and also epithelial cell impairments to lung damage and fibrosis upon CD39 deficiency.

We further analyzed if the non-canonical adenosinergic CD38/CD203a pathway might contribute to radiation-induced lung fibrosis. The expression of both receptors was restricted to only a minor group of lungs infiltrating myeloid/ macrophage-like cells, whereas resident lung cells clearly showed no immunoreactivity. These findings were in line with recent reports investigating non-canonical adenosinergic CD38/CD203a signaling within immune cells. CD203a expression was found to be restricted to plasma B cells (122). Accordingly, CD203a/PC-1 expression was found to be rather low on lymphocytes, monocytes, and granulocytes of the bone marrow (123). In contrast to CD203a, a high CD38 expression on circulating monocytes has been described (56, 124, 125). Resident lung macrophages can also express CD38 (126, 127). Both studies highlight that CD38 expression on macrophages was associated with an inflammatory M1 phenotype and chronic inflammation. Of note, in the study from Dewhurst et al., it is further described that only the small alveolar and interstitial macrophages express CD38 and that there was only a low expression on big foamy macrophages (126). We showed already that small macrophages can be distinguished from bigger foamy macrophages, which organize in clusters during fibrosis development (50). Here we revealed that only the smaller myeloid-like cells were positive for CD38. As CD38 is suggested to normal inflammatory responses in the lung toward hyperinflammation (55), we conclude that CD38 signaling on small monocytes/macrophages, with a potential M1 phenotype, is not linked to a profibrotic phenotype. However, non-canonical adenosinergic signaling might have an indirect impact on profibrotic signaling: NAD^+^/CD38 signaling was shown to influence Treg survival, phenotype, and function. CD38^−/−^-deficient mice studies revealed that extracellular NAD^+^ can deplete and reduce Treg (128). In contrast, an increase in Tregs during the fibrotic phase of radiation-induced pneumopathy already highlighted that besides M2-like macrophages also Treg might contribute to the development of lung fibrosis (49, 129, 130). Herein, extracellular Ado positively alters Treg survival and functions (131). Thus, besides CD73/Ado signaling (Ado accumulation), even CD38/CD203a signaling (NAD^+^ reduction) could led to the observed increase in Treg during the fibrotic phase (49).

## Conclusion

Genetic deficiency of CD39 in the host may result in long-term adaptive processes, e.g., in the microvasculature or the immune system, respectively, with impact on tumor growth and radiation response in tumor and normal tissues. Such adverse effects are reminiscent of suggested risks of sustained CD39 inhibition (132). Thus, a careful testing of pharmacologic strategies interfering with CD39 activity is needed to exclude that such a therapeutic strategy exerts growth- and resistance-promoting effects in tumor tissues, adverse effects in normal tissues, or both, particularly when used in combination with a single dose or clinically more relevant fractionated radiation schedules. Loss of CD39 or activation of CD73 impacts on radiation-induced changes in the lung environment, e.g., ATP/Ado ratio, as well as phenotype and function of resident cells and recruited immune cells with impact on cell survival, vascular function, immune defense, matrix deposition, and fibrosis. Of note, both CD39 and CD73 are important regulators of the TME, particularly in hypoxic tumors, with the ability to suppress immune-mediated tumor cell killing and to promote tumor growth, angiogenesis, and progression (133). Thus, targeting pathologic aspects of CD39/CD73 signaling might be an attractive approach to enhance efficacy of therapies involving RT in malignant tumors without enhancing normal tissue toxicity or to protect normal tissues from the adverse effects of RT without protecting the tumor. Further work is highly needed to unravel the multifaceted roles of purinergic signaling in the complex interactions and signaling networks between the microenvironment in tumor and normal tissues by using orthotopic lung tumor models and/or genetically engineered mouse models as well as clinically relevant fractionated radiation schedules.

## Data Availability Statement

The raw data supporting the conclusions of this article will be made available by the authors, without undue reservation.

## Ethics Statement

The animal study was reviewed and approved by Landesamt für Natur, Umwelt und Verbraucherschutz (LANUV), Regierungspräsidium Düsseldorf. Written informed consent was obtained from the owners for the participation of their animals in this study.

## Author Contributions

AM designed research, performed experiments and evaluated data, wrote, and critically reviewed manuscript. FW and DK designed research, performed experiments, analyzed data, wrote, critically reviewed, and revised the manuscript. SL and KS performed experiments and critically reviewed manuscript. MS discussed data and critically reviewed manuscript. SR provided unique reagents. VJ designed research, wrote, and critically reviewed manuscript. All authors approved the final version of the manuscript.

## Conflict of Interest

The authors declare that the research was conducted in the absence of any commercial or financial relationships that could be construed as a potential conflict of interest.
