# Supplementary material for: Host CD39 Deficiency Affects Radiation-Induced Tumor Growth Delay and Aggravates Radiation-Induced Normal Tissue Toxicity
Source: Front Oncol. 2020 Oct 22;10:554883. doi: 10.3389/fonc.2020.554883 (PMC7649817; doi:10.3389/fonc.2020.554883)
Supplement: Supplementary file 1 [file Data_Sheet_1.PDF]

Figure S1.

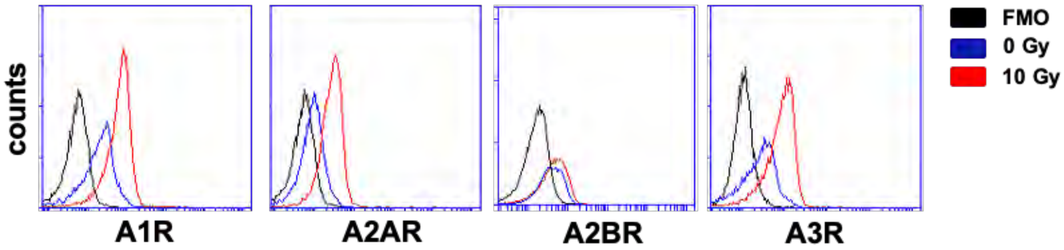

Figure S1.: Irradiation with 10 Gy increases the expression of adenosine receptors A1R, A2AR and A3R in LLC1 cells. LLC1 tumor cells were irradiated with 0 and 10 Gy, respectively. LLC1 cells were stained against A1R, A2AR, A2BR, A3R. Shown in histograms is the intensity of adenosine receptor (A1R, A2AR, A2BR, A3R) expression, analyzed by flow cytometry 24 h after irradiation. FMO = fluorescence minus one);

Figure S2.

(A)

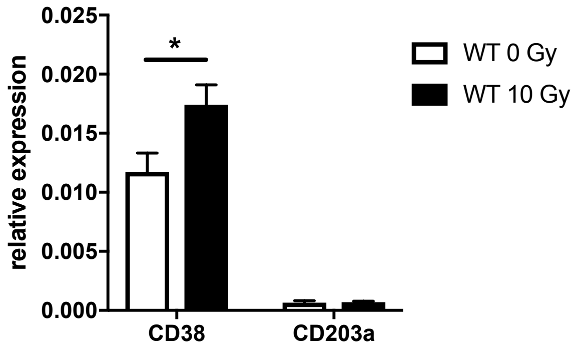

(B)

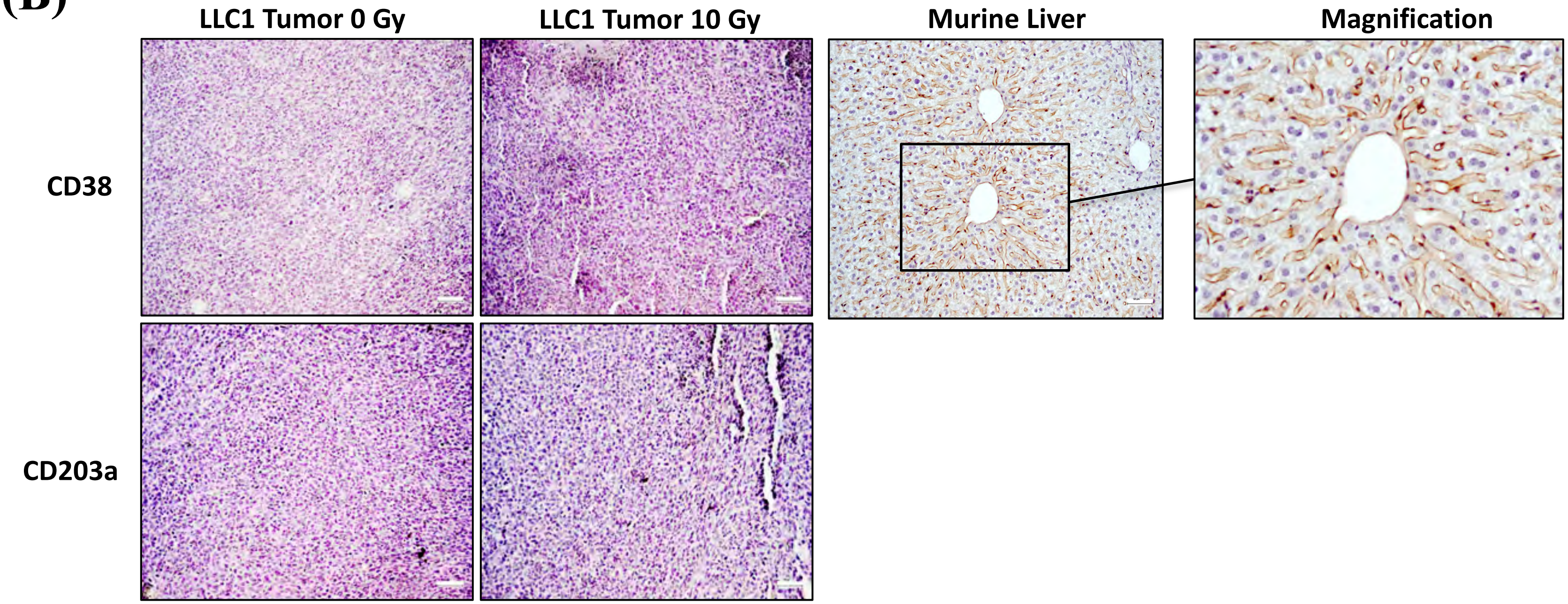

Figure S2.: LLC1 tumor grown on either WT, CD39$^{-/-}$ or CD73$^{-/-}$ are negative for CD38 and CD203a. LLC1 tumor cells (0.5 x 10^6 cells each) were subcutaneously transplanted onto the hindlimb of C57BL/6 wildtype, CD39- and CD73-deficient (knockout (-/-)) mice. Hind leg single dose irradiation with 0 or 10 Gy was conducted at the timepoint of tumor manifestation. Mice were sacrificed at the timepoint of maximal tumor volume (1000 mm^3). (A) RT qPCR analysis to reveal the relative expression of CD38 and CD203a/PC-1 to actin (n = 8/9, 7/9, 3/6). The relative expression is shown in bar diagrams. Shown are means ± SEM, * $p \leq 0.05$; one-way ANOVA followed by a post hoc Tukey's test. IHC staining of CD38 and CD203a/PC-1 of paraffin-embedded LLC1 tumor tissue from WT mice, magnification 200-fold, scale bar 50 µm. Left panel = unirradiated, middle left panel = irradiated, middle right panel = positive control liver tissue (sinusoids), right panel = magnified pictures (400 fold) highlight positive staining in sinusoids. Representative qRT-PCR data and pictures from WT mice.

Figure S3.

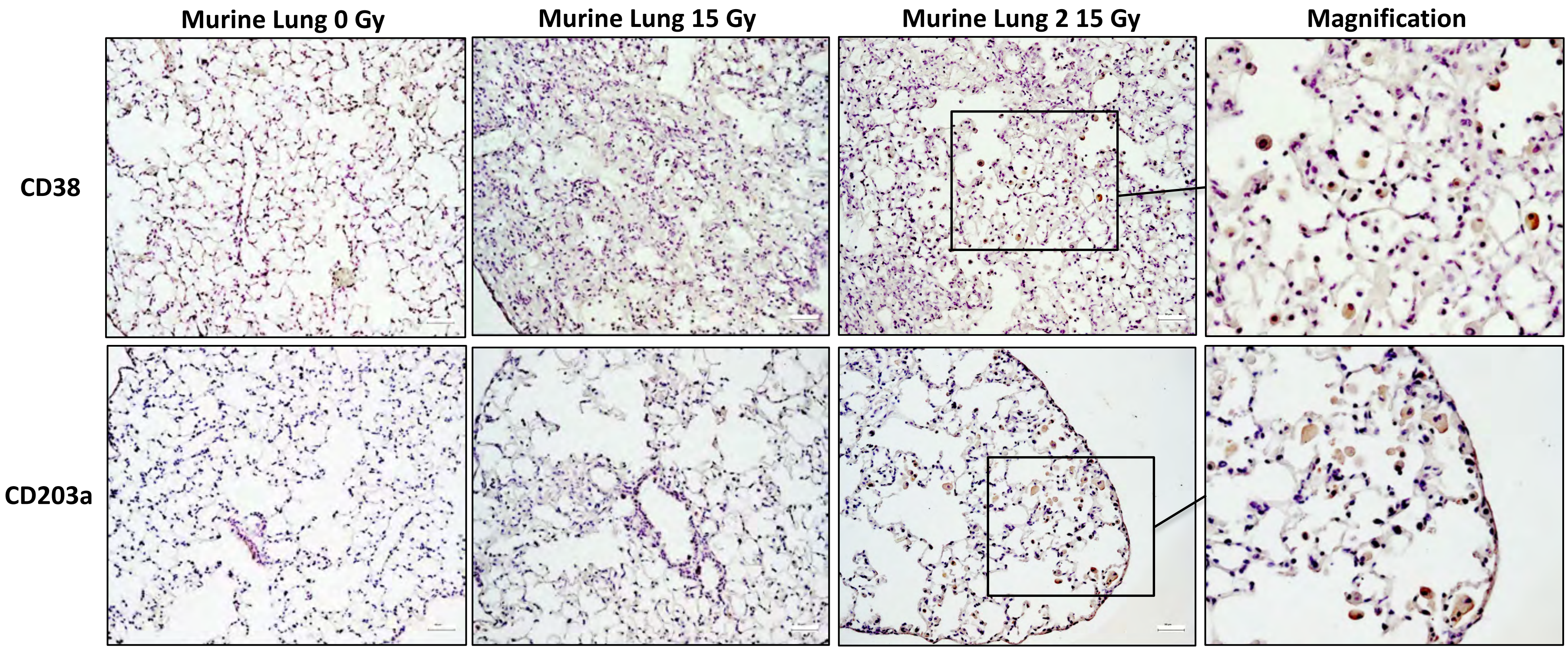

Figure S3.: Irradiated lungs from WT or CD39$^{-/-}$ mice have CD38^{+}/CD203a^{+} immune cells. C57BL/6 (WT) and CD39$^{-/-}$ mice received 0 Gy or 15 Gy whole thorax irradiation (WTI) and were sacrificed at 25 weeks post-irradiation. IHC staining of CD38 and CD203a/PC-1 of paraffin-embedded lung tissue from WT mice, magnification 200-fold, scale bar 50 µm. Left panel = unirradiated, middle left panel = irradiated/ fibrotic regions, middle right panel = irradiated/ immune cells,, right panel = magnified pictures (400 fold) highlight positive stained immune cells. Representative pictures from WT mice.
